# Recombinant RGD-disintegrin Dis*Ba*-01 blocks integrin α_v_β_3_ and impairs VEGF signaling in endothelial cells

**DOI:** 10.1186/s12964-019-0339-1

**Published:** 2019-03-20

**Authors:** Taís M. Danilucci, Patty K. Santos, Bianca C. Pachane, Graziéle F. D. Pisani, Rafael L. B. Lino, Bruna C. Casali, Wanessa F. Altei, Heloisa S. Selistre-de-Araujo

**Affiliations:** Departamento de Ciências Fisiológicas, Centro de Ciências Biológicas e da Saúde, Universidade Federal de São Carlos, Rod. Washington Luis, km 235 - SP-310 - São Carlos, São Paulo, CEP 13565-905 Brazil

**Keywords:** α_v_β_3_ integrin, VEGFR2, Cross-talk, Disintegrin, Dis*Ba*-01, Extracellular matrix, Angiogenesis

## Abstract

**Background:**

Integrins mediate cell adhesion, migration, and survival by connecting the intracellular machinery with the surrounding extracellular matrix. Previous studies demonstrated the interaction between α_v_β_3_ integrin and VEGF type 2 receptor (VEGFR2) in VEGF-induced angiogenesis. Dis*Ba*-01, a recombinant His-tag fusion, RGD-disintegrin from *Bothrops alternatus* snake venom, binds to α_v_β_3_ integrin with nanomolar affinity blocking cell adhesion to the extracellular matrix. Here we present in vitro evidence of a direct interference of Dis*Ba*-01 with α_v_β_3_/VEGFR2 cross-talk and its downstream pathways.

**Methods:**

Human umbilical vein (HUVECs) were cultured in plates coated with fibronectin (FN) or vitronectin (VN) and tested for migration, invasion and proliferation assays in the presence of VEGF, Dis*Ba*-01 (1000 nM) or VEGF and Dis*Ba*-01 simultaneously. Phosphorylation of α_v_β_3_/VEGFR2 receptors and the activation of intracellular signaling pathways were analyzed by western blotting. Morphological alterations were observed and quantified by fluorescence confocal microscopy.

**Results:**

Dis*Ba*-01 treatment of endothelial cells inhibited critical steps of VEGF-mediated angiogenesis such as migration, invasion and tubulogenesis. The blockage of α_v_β_3_/VEGFR2 cross-talk by this disintegrin decreases protein expression and phosphorylation of VEGFR2 and β_3_ integrin subunit, regulates FAK/SrC/Paxillin downstream signals, and inhibits ERK1/2 and PI3K pathways. These events result in actin re-organization and inhibition of HUVEC migration and adhesion. Labelled-Dis*Ba*-01 colocalizes with α_v_β_3_ integrin and VEGFR2 in treated cells.

**Conclusions:**

Disintegrin inhibition of α_v_β_3_ integrin blocks VEGFR2 signalling, even in the presence of VEGF, which impairs the angiogenic mechanism. These results improve our understanding concerning the mechanisms of pharmacological inhibition of angiogenesis.

**Electronic supplementary material:**

The online version of this article (10.1186/s12964-019-0339-1) contains supplementary material, which is available to authorized users.

## Background

Angiogenesis, the development of new capillaries from preexisting blood vessels, is an essential process in the regulation of several physiological and pathological processes. Inadequate balance between pro-angiogenic and anti-angiogenic factors may lead to pathological conditions, notably in tumor development and metastasis. Moreover, a range of non-neoplastic diseases could be classified as ‘angiogenesis-dependent diseases’ such as diabetic retinopathy, rheumatoid arthritis, atherosclerosis and various inflammation diseases [1, 2].

Tumors exhibit considerable variation in the pattern and properties of angiogenic blood vessels, as well as in their responses to anti-angiogenic therapy. Angiogenic programming is a multidimensional process regulated by tumor cells in conjunction with a variety of tumor associated stromal cells such as cancer associated fibroblasts (CAFs) and tumor associated macrophages (TAMs), and their bioactive products, which encompass cytokines, growth factors, extracellular matrix and their ligands [3, 4]. Tumor angiogenesis is predominantly driven by vascular endothelial growth factor (VEGF), a proangiogenic growth factor expressed by many solid cancers. VEGF stimulates angiogenesis through VEGF receptor-2 (VEGFR2), a tyrosine kinase receptor expressed by endothelial cells [5–8]. VEGF-A/VEGFR2 signaling stimulates a myriad of intracellular signaling pathways such as activation of phosphatidylinositol 3-kinase (PI3K), extracellular-signal-regulated kinase (Erk) pathway, focal adhesion kinase (FAK), c-Src family and paxillin [9, 10]. The activation of such pathways results in a wide range of cell responses including increased vessel permeability and remodeling, endothelial cell proliferation, migration, tubulogenesis, secretion of matrix metalloproteinases (MMPs), nitric oxide (NO) and prostanoid synthesis [9, 11]. This synchronized signaling network, also associated with cross talking to integrin receptors, modulates the angiogenic response and it is fundamental for tumor blood supply and growth [12, 13]. Recent work demonstrated that the angiogenic switch depends on the association of a set of receptors and accessory proteins that includes αvβ3 integrin, vascular endothelial (VE)-cadherin, and syndecan-1 (sdc-1), in addition to VEGFR2 [8]. Blockade of each one of these components will affect angiogenesis in some way, which contributes to the complexity of angiogenesis control. In fact, a direct association between the cytoplasmic tails of β3 integrin and of VEGFR2 was demonstrated [14], including crosslinks mediated by the transglutaminase Factor XIII (FXIII) of the coagulation cascade [7].

Integrins comprise a family of heterodimeric transmembrane receptors that mediate cell-cell and cell-extracellular matrix (ECM) interactions, regulating cell survival, proliferation, adhesion and migration [15, 16]. Some members of the integrin family are present in endothelial cells, such as α_2_β_1_, α_5_β_1_, α_v_β_3_, and α_v_β_5_ integrins. These receptors are up-regulated during vascular remodeling and growth associated with inflammation, wound healing, ischemic injury, tumor growth and metastasis [15, 16]. The α_v_β_3_ integrin is one of the most abundant key receptor regulating angiogenesis in endothelial cells due to its cross-talk with VEGFR2 [17, 18]. Phosphorylation of VEGFR2 is enhanced when endothelial cells are plated on ECM proteins such as vitronectin and fibrinogen, which are ligands for integrin α_v_β_3_ [19]. Moreover, functional interconnections were demonstrated between α_v_β_3_ integrin and VEGFR2, resulting in up-regulation of the ligand-induced tyrosine kinase receptor activity by integrin engagement [20–22]. In vitro experiments have shown that α_v_β_3_ integrins are up-regulated by VEGF-A in microvascular endothelial cells, alongside its elevated levels at active angiogenic sites [23, 24]. Understanding how to target these cross-talk events could improve the effectiveness of current pro- or anti-angiogenic strategies.

Antibodies to integrins and small inhibitors such as RGD cyclic peptides successfully prevent angiogenesis by inhibiting ligand binding to the integrin with a subsequent blockage of adhesive functions of α_v_β_3_ integrin [25–32]. A family of small cysteine-rich proteins, mostly having the adhesive RGD motif, called disintegrins, were described from snake venom sources [33]. The RGD-disintegrins are potent antagonists of some integrins such as α_v_β_3_ and α_5_β_1_ and have anti-tumor and anti-angiogenic actions [33, 34]. Dis*Ba*-01, a recombinant His-tag fusion, RGD-disintegrin from *Bothrops alternatus* snake venom, has shown high-affinity towards α_v_β_3_ and α_IIb_β_3_ integrins, leading to strong anti-platelet and anti-thrombotic effects [35, 36]. Furthermore, Dis*Ba*-01 decreases the migration speed and directionality of oral carcinoma cells [37] and decreases VEGF receptors expression in HMEC-1 cells (Human Microvascular Endothelial Cells) [38]. In addition, in vivo assays showed that Dis*Ba*-01 inhibited both angiogenesis in two distinct animal models [35, 36, 39].

Considering the therapeutic potential of disintegrins in angiogenesis inhibition and that there is no further data on the signaling effects of this class of peptides on the cross-talk between VEGFR2 and α_v_β_3_, more in-depth knowledge is required concerning the molecular mechanisms of the action of Dis*Ba*-01 in the α_v_β_3_/VEGFR2 impairment. Here we showed for the first time, a new anti-angiogenesis mechanism of Dis*Ba*-01 by down-regulation of α_v_β_3_/VEGFR2 cross-talk and signaling. Our results help to achieve a better comprehension of the role of disintegrins in angiogenesis and may help design new tools for antiangiogenic therapy.

## Materials and methods

### Dis*Ba*-01 expression and purification

Recombinant disintegrin Dis*Ba*-01, a His-tag protein (GenBank accession AY259516) was produced from a cDNA venom gland library of a *Bothrops alternatus* snake, as previously described [35]. Strains of *Escherichia coli* BL21 (DE3) were transformed with a pET28-a plasmid containing the Dis*Ba*-01 gene. Bacterial liquid culture was grown before expression assays were performed. Cell lysis extract was purified in a three-step chromatographic process, using an affinity column (HIS-Select® Nickel Affinity Gel, Sigma-Aldrich®), followed by a size-exclusion chromatography (Superdex 75 10/300 GL, GE Healthcare) and an anion exchange column (Mono-Q 5/50 GL, GE Healthcare). Total protein was determined by colorimetric detection of bicinchoninic acid assay (Pierce™ BCA Protein Assay, Thermo Scientific). Recombinant human VEGF_165_ was from Peprotech.

### Cell culture

Human umbilical vein endothelial cells (HUVEC, American Type Culture Collection [ATCC® CRL-1730]) were cultivated in Dulbecco’s Modified Eagle Medium (DMEM, Vitrocell) supplemented with 10% fetal bovine serum (FBS, Nutricell), penicillin (10.000 U.I./mL) and streptomycin (10 mg/mL) (Vitrocell). Cells were maintained incubated at 37 °C, on atmosphere with 5% CO_2_. Subcultures of cells were performed as instructed by the supplier, using trypsin-EDTA. Cells were used between passages 8 to 15 and counted on a TC20 automated cell counter (Bio-Rad) using trypan blue stain solution at 0.4% (Thermo Scientific).

### Cell viability assay

Cell viability or possible cytotoxicity of treatments was tested in a 96-well plate, where HUVECs (5 × 10^3^ cells/well) were plated on serum-supplemented medium and left to adhere for 24 h on incubator. A 24-h starvation period on serum-free DMEM occurred, followed by the treatment of cells with Dis*Ba*-01 (1000 nM) and/or VEGF (10 ng/ml; PeproTech)). Cells were cultured for 24 h at 37 °C, 5% CO_2_. Viable cells were identified using MTT solution (0.5 mg/ml of 3-(4,5-Dimethylthiazol-2-yl)-2,5-Diphenyltetrazolium Bromide, ThermoFisher Scientific) for 3.5 h. Samples were diluted in isopropanol for measurement of cell concentration by spectrophotometry SpectraMax i3x (OD_540 nm_, Molecular Devices).

### Cell invasion assay

Cell invasion was tested using a 24-well plate Matrigel™ invasion chamber (Corning) previously hydrated with serum-supplemented DMEM. HUVECs (2 × 10^5^ cells/well) were treated with 1000 nM Dis*Ba*-01 and/or VEGF (10 ng/ml) on serum-free DMEM medium for 30 min at 4 °C. Cells were pipetted into the Boyden’s chamber whilst it was inserted on well containing DMEM 10% FBS. The negative control comprised of serum-free DMEM on the wells. Invasion was allowed to occur for 18 h at 37 °C. After that, samples were fixed in 4% paraformaldehyde and cell nuclei were stained with DAPI (0.7 ng/μl). Using Vectashield® mounting media (Vector Laboratories), membranes were assembled into slides for cell counting on automated fluorescence microscope system, ImageXpress Micro (Molecular Devices).

### Transwell migration assay

The ability of cells to migrate was tested in a transwell assay, using a ThinCert™ translucent PET membrane RoTrac®, 8.0 μm pore (Greiner Bio-one®). HUVECs (1 × 10^5^ cells/well) were exposed to Dis*Ba*-01 (1, 10, 100 and 1000 nM), VEGF (10 ng/ml) or VEGF plus Dis*Ba*-01 (1000 nM) and immediately inserted into the Boyden’s chamber. The chambers were immersed in 10% FBS medium and allowed to migrate for 6 h at 37 °C. As negative control, chambers were inserted on serum-free medium and incubated as indicated above. Migrated cells were fixated on the membranes with 4% paraformaldehyde and its nuclei were stained in DAPI solution (0.7 ng/μl). Membranes were assembled in histological slides using Vectashield® mounting media (Vector Laboratories) for automated cell counting on ImageXpress Micro microscope (Molecular Devices).

### Inhibition of adhesion

HUVECs inhibition of adhesion to fibronectin (FN) and vitronectin (VN) was determined in an assay using a 96-well black plate whose wells were pre-coated with either solution (1 μg/cm^2^ (FN) and 0.2 μg/cm^2^ (VN), Sigma-Aldrich). The negative control comprised coating of 2% BSA. Non-specific binding was blocked with 1% BSA for 1 h at 37 °C, 5% CO_2_. After its removal, wells were washed with PBS. Meanwhile, HUVECs (1 × 10^5^ cells/well) were treated with Dis*Ba*-01 (1000 nM) and/or VEGF (10 ng/ml) and immediately seeded in their respective wells. Cells were allowed to adhere for 1 h at 37 °C, 5% CO_2_. Wells were extensively washed with PBS before fixation using 4% paraformaldehyde solution (pH 7.5) for 15 min. Cell nuclei were stained with DAPI (0.7 ng/μl) and counted automatically using ImageXpress (Molecular Devices).

### Endothelial cell tube formation assay

Tubulogenesis assay on Matrigel was performed to evaluate the effect of Dis*Ba*-01 on HUVECs tube formation. According to the manufacturer’s instructions, Matrigel solution (Corning® Matrigel® Basement Membrane Matrix, *LDEV-Free) was thawed in a refrigerator at 4 °C overnight. Wells of a pre-cooled 96-well plate was coated with Matrigel (35 μl/well), followed by immediate placement in a humidified CO_2_ incubator at 37 °C for 1 h for coating solidification. HUVECs (3 × 10^4^ cells/well) were treated for 30 min with VEGF (10 ng/ml, PeproTech), Dis*Ba*-01 (1, 10, 100 and 1000 nM) or VEGF plus Dis*Ba*-01 (1000 nM) in DMEM containing 0.5% FBS and then seeded on the solidified Matrigel. The plate was placed in a humidified CO_2_ incubator at 37 °C for 14 h to allow the formation of tubes. Images were photographed using the AxionVision Rel.4.8 software of a Vert.A1 microscope (Zeiss) and analysed using the Angiogenesis Analyser plugin for ImageJ software (version 1.51n).

### Analysis of gene expression by quantitative PCR

RNA extraction started with the plating of HUVECs (5 × 10^5^/well) in 6-well plates with DMEM 10% FBS, followed by a 24-h starvation period on serum-free medium. Cells were treated with Dis*Ba*-01 (1000 nM) and/or VEGF (10 ng/ml) for 24 h and collected for RNA extraction using TRIZOL reagent (Invitrogen). Total RNA was extracted according to the manufacturer’s instructions. RNA pellet was resuspended in nuclease-free water and stored at − 80 °C. Nanodrop 2000 (Thermo Scientific) was used for measuring the RNA concentration and purity (260/280 nm and 260/230 nm ratios). RNA (1 μg) was treated with deoxyribonuclease I, Amplification Grade (Invitrogen) and *iScriptTM cDNA Synthesis* (BioRad Laboratories) was used for reverse transcription according to the manufacturer’s specifications. CFX 96 real-time PCR detection system (Bio-Rad) was used for qPCR reaction. Each reaction used 20 ng of cDNA, 400 nM of each primer and 5 μl of SsoFast™ Evagreen Supermix (Bio-Rad) in a total volume of 10 μl per reaction. Gene specific primers: KDR sense primer (5′-3′) GTACATAGTTGTCGTTGTAGG antisense primer (3′-5′) TCAATCCCCACATTTAGTTC (Sigma-Aldrich); ITGB-3 sense primer (5′-3′) CTCCGGCCAGAATCC antisense primer (3′-5′) TCCTTCATGGAGTAAGACAG (Sigma-Aldrich) and GAPDH sense primer (5′-3′) GACTTCAACAGCGCGACACCCAC antisense primer (3′-5′) CACCACCCTGTTGCTGTAG (Exxtend). The thermal cycling program was set for 10 min at 95 °C, followed by 40 cycles of 15 s at 95 °C, 30 s at 60 °C and 30 s at 72 °C. After the run, the melting curve was analysed to confirm the specificity of the amplification products. GAPDH was used as a housekeeping gene. The relative expression of qRT-PCR products was determined through ΔΔCt method, in which relative expression was calculated using the following equation: fold induction = 2 ^–ΔΔCt^ [40].

### Flow cytometry

HUVECs (5 × 10^5^/well) were seeded in 6-well plates with DMEM 10% FBS, followed by a 24-h starvation period on serum-free medium. Cells were treated with Dis*Ba*-01 (1000 nM) and/or VEGF (10 ng/ml) for 24 h. The characterization of β_3_ integrin in HUVECs was measured by flow cytometry using specific fluorescent-labelled antibodies. Cells (1 × 10^6^) were incubated with 1 mg of anti-integrin β_3_ antibody (human anti-mouse, 1 μg, Santa Cruz) at 4 °C for 40 min, followed by wash with PBS and centrifugation at 4 °C for 10 min at 1300 rpm. Then, 0.5 mg of secondary antibody (goat anti-mouse IgG, Biosciences BD) labelled with the fluorophore FITC (Fluorescein Isothiocyanate, 2.5 μl/tube) was added to each sample and incubated for 45 min at 4 °C in the dark. Cells were washed with PBS, centrifuged and analysed with Accuri flow cytometer (BD Biosciences).

### Western blot

HUVECs (5 × 10^5^ cells/well) were seeded in 6-well plates and left to adhere on an incubator at 37 °C, 5% CO_2_, overnight, followed by a period of 24 h of starvation at serum-free medium. Cells were treated with 1 ml of DMEM supplemented with 10% FBS and either Dis*Ba*-01 (1000 nM), VEGF (10 ng/ml; PeproTech) or a co-treatment and incubated for 1 and 24 h at 37 °C, 5% CO_2_. Cell lysis was performed using 100 μl of lysis buffer (50 mM Tris-HCl pH 7.4, 150 nM NaCl, 1 mM EDTA, 1 mM sodium orthovanadate, 1 mM sodium fluoride, 1% Tween 20, 0.25% sodium deoxycholate, 0.1 mM phenylmethylsulfonyl fluoride, 1 μg/ml aprotinin and 1 μg/ml leupeptin) and the cell lysates were centrifuged at 14000 g, 4 °C for 20 min. Protein content of the supernatant was determined by a BCA Protein Assay Kit (Thermo Fisher Scientific). Cellular proteins (20 μg) were separated on a 20% SDS-PAGE, transferred to nitrocellulose membranes (0.45 μm; Bio-Rad) and blocked with Tween-TBS buffer (140 mM NaCl, 2.6 mM KCl, 25 mM Tris, pH 7.4, 0.05% Tween 20) plus 5% powdered milk. Western Blot was performed using the antibodies anti-phospho-ERK1 + ERK2^Y187^ (1:500; Abcam ab47339), anti-phospho-PI3K^Y607^ (1:1000; Abcam ab182651), anti-VEGFR2 (1,5 μg/ml; Abcam ab39256), anti-phospho-VEGFR2^Y1054 + Y1059^ (0.5 μg/ml; Abcam ab5473), anti-phospho-Src^Tyr418^ (1:1000; Abcam), anti-phospho-paxillin (1:1000; Abcam), anti-phospho-β_3_^Y773^ (1:1000; Abcam ab38460) and anti-phospho-FAK^Y397^ (1:1000; Abcam ab40794) and revealed with a Chemiluminescent Reagent (Sigma Aldrich). After that, membranes passed through stripping and GAPDH (1:1000; Abcam) was used as housekeeping antibody. Bands were visualized on a molecular imager (ChemiDoc™ XRS; Bio-Rad). At least three experiments in triplicate were performed for each protein and the bands were quantified by densitometric analysis using ImageJ FIJI program.

### Morphological analysis

HUVECs (3 × 10^4^ cells/well) were plated in a 96-well Microplate μClear® Black CellStar® (Greiner bio-one), previously coated with fibronectin (1 μg/ml), in serum-free DMEM and incubated overnight at 37 °C, 5% CO_2_. Cells were exposed to VEGF (10 ng/ml), Dis*Ba*-01 (1000 nM) and VEGF plus Dis*Ba*-01 for 30 min in DMEM 10% FBS. Afterwards, cells were fixed in 4% paraformaldehyde for 20 min, blocked for 1 h with 1% BSA and incubated with 0.7 μg/ml DAPI (Thermo Fisher Scientific) and Alexa Fluor™ 488 phalloidin (Life Technologies) for 10 min. Fluorescent samples were observed using ImageXpress (Molecular Devices) equipment with 60x magnification.

### Co-localization assay

HUVECs (5 × 10^4^ cells/well) were plated in glass coverslips, previously coated with fibronectin (1 μg/cm^2^), in serum-supplemented DMEM and left overnight in an incubator at 37 °C, 5% CO_2_. Dis*Ba*-01 (1000 nM), previously labelled using Alexa Fluor® 546 dye (Invitrogen, Thermo Scientific), was added to the cells for 2 min. Samples were fixed in 4% paraformaldehyde for 10 min and permeabilized using 0.5% Triton X-100 for 10 min. Samples were washed with PBS, followed by a 1-h incubation in 5% PBS-BSA to block unspecific sites. Cells were incubated overnight with targeted primary antibodies (1:100 Rabbit pAb to VEGF Receptor 2; 1:100 Mouse Monoclonal to the integrin α_v_β_3_, Abcam). Then, secondary antibodies (1:1000 Alexa Fluor 633 goat anti-rabbit, ThermoFisher; 1:1000 Goat polyclonal anti-mouse Alexa Fluor 488, ThermoFisher Scientific) were mixed in 5% PBS-BSA and applied on the wells. After incubation, slides were cleaned and samples were stained with DAPI (Thermo Fisher Scientific) for 10 min. Slides were assembled using ProLong™ Antifade Reagents for Fixed Cells (Thermo Fisher Scientific) and observed on confocal microscope Axio Observer LSM 780 (Zeiss) aided by ZEN BLACK software. Analysis occurred under the same laser intensity for different fluorescences at 63x magnification. Colocalization coefficients were determined using ImageJ FIJI program.

### Statistical analysis

Data were obtained from at least three independent series of experiments and analyses were performed using the statistical program GraphPad Prism (version 5.0). Data were expressed as mean ± standard error of the mean (SEM) and intergroup comparisons were made using One-way ANOVA with Bonferroni as post hoc and t test (parametric). Values of *p* < 0.05 were considered statistically significant.

## Results

### Dis*Ba*-01 blocks several critical steps in angiogenesis

**Fig. 1:**
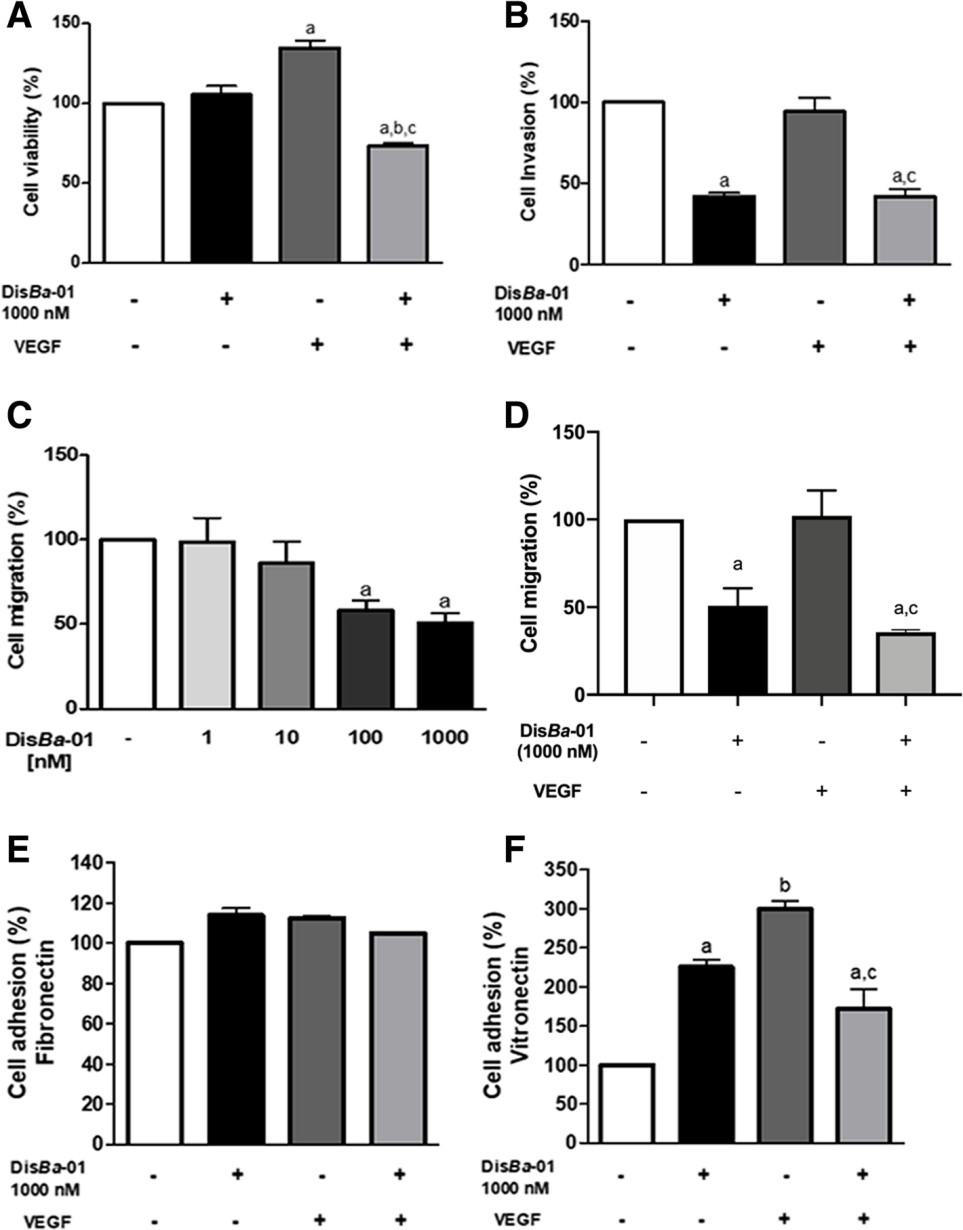
Dis*Ba*-01 effects on VEGF-induced HUVEC viability, invasion, migration and adhesion. **a** Cells were treated with Dis*Ba*-01 (1000 nM), VEGF (10 ng/mL) or both proteins in DMEM supplemented with 0.5% FBS followed by 24 h of incubation. Cell viability was measured by spectrophotometry at 540 nm after incubation with MTT. **b** HUVECs (2 × 10^5^ cells/well) were treated with 1000 nM Dis*Ba*-01 and/or VEGF (10 ng/mL) on serum-free DMEM for 30 min at 4 °C. Cells were pipetted into the Boyden’s chamber and then it was inserted on well containing DMEM 10% FBS. The negative control comprised of serum-free DMEM on the wells. Invasion was allowed to occur for 18 h at 37 °C. Cell nuclei were stained with DAPI (0.7 ng/μl). Quantification of invasive cells was measured by automated cell counting. **c-d** For the migration assay, HUVECs (1 × 10^5^ cells/well) were exposed to Dis*Ba*-01 (1, 10, 100 and 1000 nM), VEGF (10 ng/mL) or VEGF plus Dis*Ba*-01 (1000 nM) and immediately inserted into the Boyden’s chamber. The chambers were immersed in 10% FBS medium and allowed to migrate for 6 h at 37 °C. Control chambers were inserted in serum-free medium. Cell nuclei were stained with DAPI (0.7 ng/μl) and cell migration was measured by automated cell counting. **e-f** HUVECs (1 × 10^5^ cells/well) were treated with Dis*Ba*-01 (1000 nM) and/or VEGF (10 ng/mL) and were immediately incubated (37 °C, 1 h) in fibronectin and vitronectin precoated-wells. Negative control was comprised of wells coated with 2% BSA. Cell nuclei were stained with DAPI (0.7 ng/μl) and quantification of adhesion cells was measured by automated cell counting. Results represent the average of three independent experiments in triplicate. Values of *p < 0.05 were significantly different when compared to untreated (a), treated with Dis*Ba*-01 (b), or with VEGF (c)

Four different experiments were designed to explore how Dis*Ba*-01 could inhibit angiogenesis. Therefore, we stimulated HUVECs with VEGF, treated with Dis*Ba*-01 and analysed the changes on proliferation/viability, migration, invasion and adhesion of HUVEC to ECM components. The number of viable endothelial cells was increased by VEGF (34.5%) after a 24-h incubation period as expected, and Dis*Ba*-01 alone had no effect on cell viability (Fig.1a). However, Dis*Ba*-01 significantly inhibited VEGF-induced proliferation by 61%. HUVEC matrigel invasion was significantly inhibited (58%) by Dis*Ba*-01 after 18 h (Fig. 1b) even in the presence of VEGF, which had no effect in this assay. Cells exposed to Dis*Ba*-01 (100 and 1000 nM), but not to VEGF, exhibited a significant decrease in cell migration (43 and 49%, respectively) (Fig. 1c). VEGF treatment did not affect cell migration; however, the inhibitory effect of DisBa-01 was higher (69%) in the presence of VEGF (Fig. 1d).

Integrin α_v_β_3_ is a multifunctional receptor that binds to at least four RGD-containing adhesive proteins, including FN and VN. We sought to determine how Dis*Ba*-01 interferes with HUVEC adhesion to FN and VN. Neither the disintegrin or the growth factor affected cell adhesion to FN (Fig. 1e). However, both DisBa-01 and VEGF increased HUVEC adhesion to VN (Fig. 1f). Interestingly, HUVEC adhesion was lower (41%) when HUVECs were simultaneously treated with the two proteins (Fig. 1f). These results support the hypothesis that the interaction of Dis*Ba*-01 with endothelial cell surface receptors prevents VEGF-induced cell proliferation and adhesion to VN.

### Dis*Ba*-01 inhibits HUVEC tubulogenesis

**Fig. 2:**
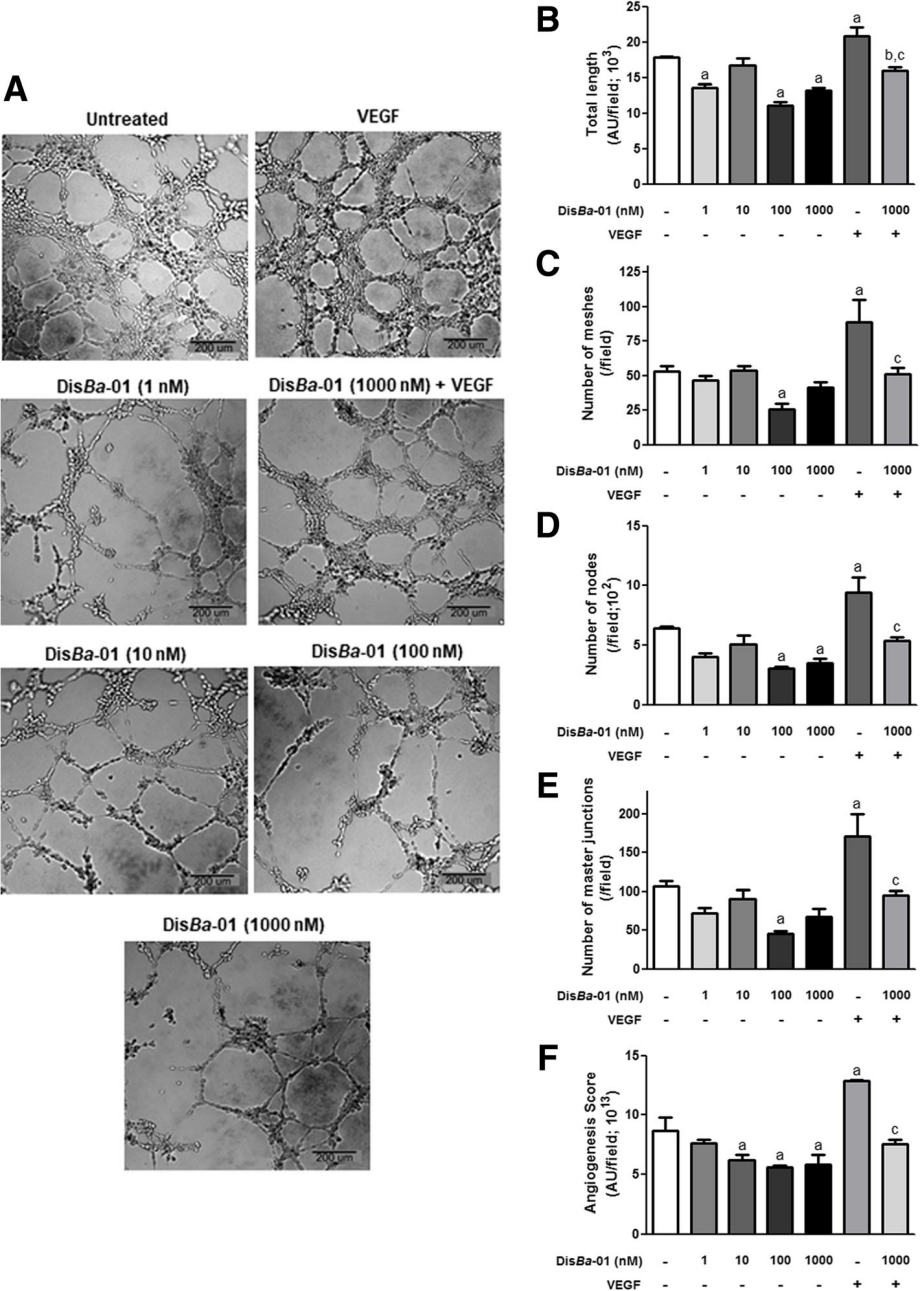
Dis*Ba*-01 inhibits HUVEC tubulogenesis. HUVECs (3 × 10^4^ cells/well) were treated for 30 min with VEGF (10 ng/mL), Dis*Ba*-01 (1, 10, 100 and 1000 nM) or VEGF plus Dis*Ba*-01 (1000 nM) in DMEM containing 0.5% FBS and then seeded on a solidified Matrigel. The plate was placed in a humidified CO_2_ incubator at 37 °C for 14 h to allow the formation of tubes. **a** Photos (40x magnification) were obtained from a representative experiment (*n* = 3). The results were expressed as **b** Total length (μm^2^), **c** Number of mashes, **d** Number of nodes, **e** Number of master junctions and **f** Angiogenesis Score (analysed area x tube length x total of branches). Images were photographed using the AxionVision Rel.4.8 software of a Vert.A1 microscope (Zeiss) and analysed using the Angiogenesis Analyzer plugin for ImageJ software (version 1.51n). Results represent the average of three independent experiments in triplicate. Values of *p < 0.05 were significantly different when compared to untreated (a), Dis*Ba*-01 (b) and VEGF (c) groups

The formation of tubes is a critical step in angiogenesis and therefore, we tested whether Dis*Ba*-01 would interfere in HUVEC tubulogenesis induced by VEGF. HUVEC growth on Matrigel generated a stabilized network of capillary-like structures, as demonstrated by the complexity of the tubular network per field in untreated and VEGF-stimulated cells (Fig. 2). VEGF treatment increased the tube total length (17%), the number of meshes (67%), nodes (47%), master junctions (60%) and the angiogenesis score as expected (Fig. 2a-f).

Next, we tested the effects of Dis*Ba*-01 on VEGF angiogenic action. Dis*Ba*-01-treated cells produced tubes morphologically distinct from both untreated and VEGF-treated cells (Fig. 2a). The disintegrin also decreased the total tube length (23% at 1 nM; 38% at 100 nM and 26% at 1000 nM) and at the highest concentration (1000 nM) Dis*Ba*-01 abolished the stimulatory effect of VEGF, resulting in equal values of control cells (Fig. 2b). Similar results were observed for the number of capillary-like mesh structures, number of nodes and master junctions (Fig. 2c-e). Furthermore, we calculated the angiogenesis score (analysed area x tube length x total of branches) which indicated that Dis*Ba*-01, in most tested concentrations, inhibited tube formation (56%, Fig. 2f) and the VEGF effect (40%, Fig. 2f). These results demonstrate that Dis*Ba*-01, at least at 1000 nM, negatively modulated VEGF angiogenic effects. We did not test the effect of lower concentrations of Dis*Ba*-01 plus VEGF.

### Dis*Ba*-01 inhibits VEGFR2 but not β_3_ expression

Attempts to elucidate the mechanisms of inhibition of angiogenesis by Dis*Ba*-01 included the analysis of VEGF receptors and β_3_ integrin subunit expression in vascular endothelial human cells under VEGF and Dis*Ba*-01 treatments. Dis*Ba*-01 does not affect β_3_ protein expression as determined by flow cytometry (Additional file 1: Figure S1A). In addition, Dis*Ba*-01 treatment did not affect mRNA levels of β_3_ integrin subunit in either VEGF-treated and untreated groups (Additional file 1: Figure S1B).

**Fig. 3:**
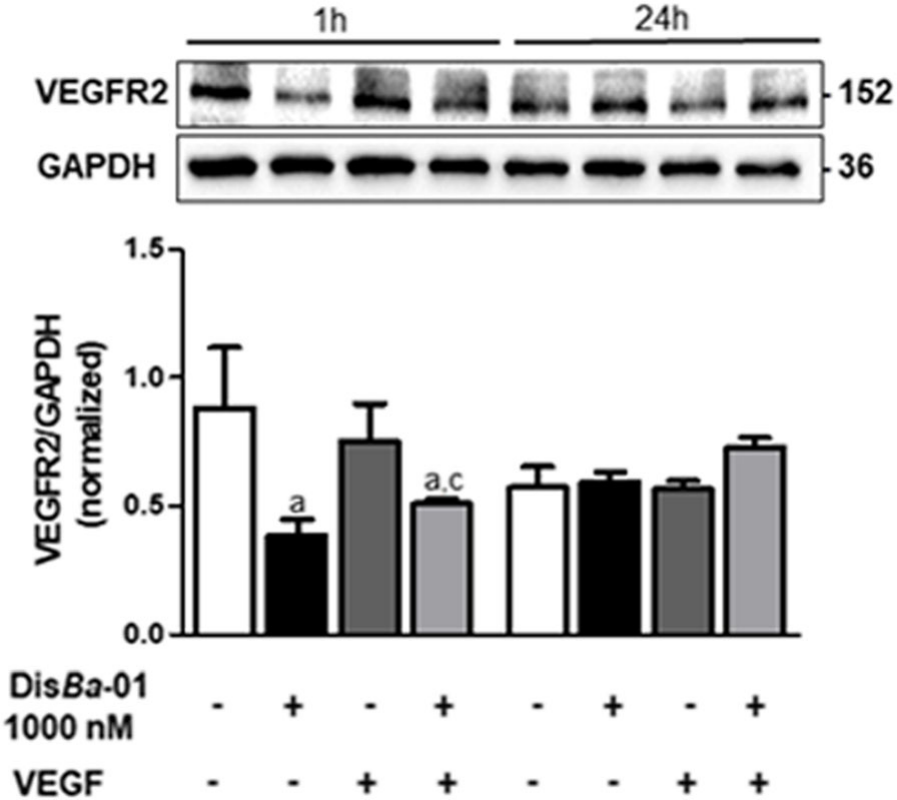
Dis*Ba*-01 decreases VEGFR2 protein content. **a** Analysis of VEGFR2 protein content by western blot. HUVECs (5 × 10^5^ cells/well) were seeded in 6-well plates and left to adhere on an incubator at 37 °C, 5% CO_2_, overnight, followed by a period of 24 h of starvation at serum-free medium. Cells were treated with 1 ml of DMEM supplemented with 10% FBS and either Dis*Ba*-01 (1000 nM), VEGF (10 ng/mL) or a co-treatment and incubated for 1 and 24 h at 37 °C, 5% CO_2_, followed by cell lysis. Twenty micrograms of protein from cell lysates were separated on SDS-PAGE. Blots were probed with VEGFR2 antibody and GAPDH antibody was used to normalize analysis. Bands corresponding to all proteins were quantified by densitometry using the ImageJ FIJI program. Bar graph shows the mean ± SE of VEGFR2/GAPDH expression from three independent experiments. **b** VEGFR2 mRNA (KDR) expression. HUVECs (5 × 10^5^/well) were seeded in 6-well plates containing DMEM and 10% FBS, followed by a 24-h starvation period on serum-free medium. Cells were treated with Dis*Ba*-01 (1000 nM) and/or VEGF (10 ng/mL) for 24 h followed by lysis and RNA isolation. Quantitative RT-PCR was carried out using specific primers to human KDR (VEGFR2) and GAPDH (housekeeping). Bar graph shows the mean ± SE of VEGFR2 expression from three independent experiments. Values of *p < 0.05 were significantly different when compared to untreated (a), Dis*Ba*-01 (b) and VEGF (c) groups

Dis*Ba*-01 down-regulated VEGFR2 protein expression in cell lysates after 1 h of exposure when compared to the VEGF-stimulated and unstimulated groups (Fig. 3a). VEGFR2 levels were back to normal after 24 h. VEGF treatment alone did not affect VEGFR2 protein expression. Furthermore, Dis*Ba*-01 treatment did not affect mRNA levels of VEGFR2 (Fig. 3b) in both VEGF-treated and untreated groups.

### Dis*Ba*-01 impaired VEGFR2 and β_3_ cross-talk

**Fig. 4:**
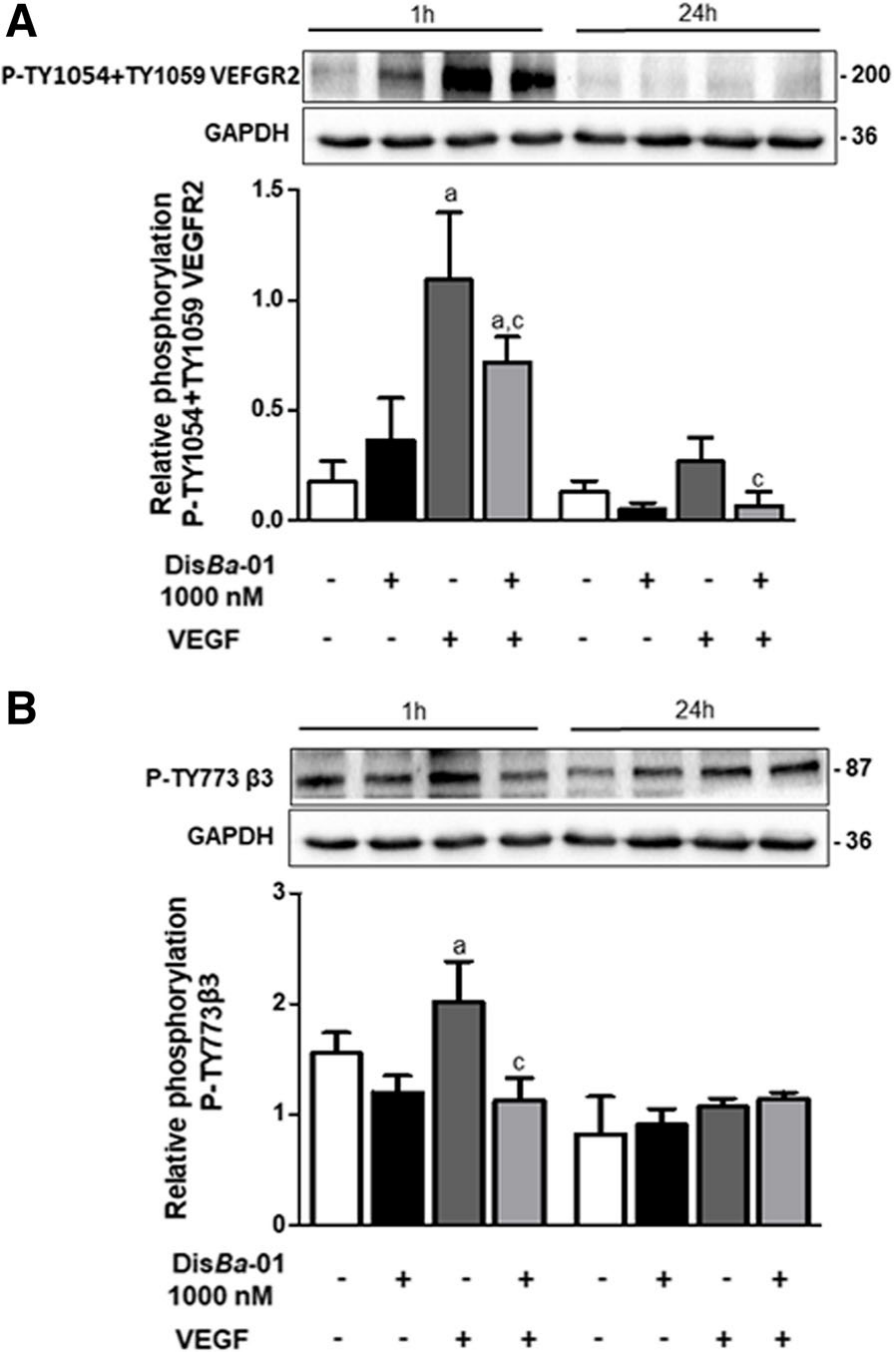
Dis*Ba*-01 inhibits VEGFR2 and β_3_ phosphorylation after VEGF stimulation. HUVECs (5 × 10^5^ cells/well) were seeded in 6-well plates and left to adhere at 37 °C, 5% CO_2_, overnight, followed by a period of 24 h of starvation in serum-free medium. Cells were treated with 1 ml of DMEM supplemented with 10% FBS and Dis*Ba*-01 (1000 nM), VEGF (10 ng/mL) or a co-treatment and incubated for 1 and 24 h at 37 °C, 5% CO_2_, followed by cell lysis. Twenty micrograms of protein from cell lysates were resolved by SDS-PAGE. Blots were probed with antibodies to **a** P-TY1054 + TY1059 VEGFR2, to **b** P-Ty773β_3_ and GAPDH, this last to normalize loading. Bands corresponding to all proteins were quantified by densitometry using the ImageJ FIJI program. Bar graph shows the mean ± SE of phosphorylated VEGFR2/GAPDH and β_3_/GAPDH expression from three independent experiments. Values of *p < 0.05 were significantly different when compared to untreated (a), Dis*Ba*-01 (b) and VEGF (c) groups

The interaction between β_3_ and VEGFR2 occurs in synergism. The signalling for VEGFR2 phosphorylation is originated on β_3_ phosphorylation, a process initiated by VEGFR2 activation after its ligation to VEGF [20]. We evaluated whether Dis*Ba*-01 could interfere in this cross-talk. VEGFR2 phosphorylation was significantly increased under VEGF stimulation; however, this effect was inhibited by 1000 nM Dis*Ba*-01 (Fig. 4a). Likewise, as shown in Fig. 4b, VEGF also induced β_3_ phosphorylation, which was also reversed by Dis*Ba*-01. These data suggest that Dis*Ba*-01 impaired the cross-talk between α_v_β_3_ and VEGFR2, which is crucial for angiogenesis regulation. β_3_ phosphorylation induced by VEGF was observed only at 1 h after the treatments, and was normalized after 24 h.

### The anti-angiogenic effect of Dis*Ba*-01 is sustained by ERK1/2 inhibition

**Fig. 5:**
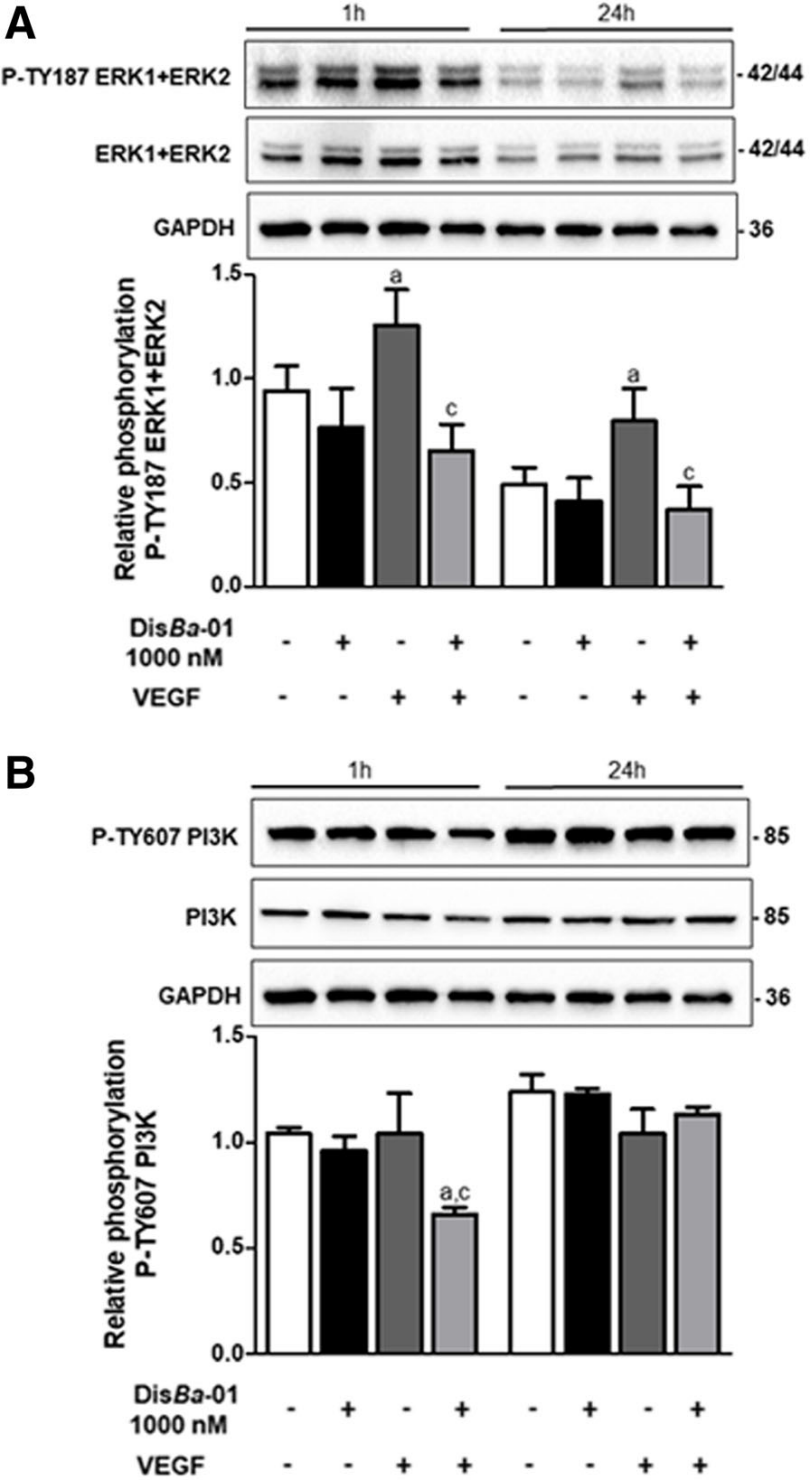
Dis*Ba*-01 inhibits ERK1/2 and PI3K phosphorylation. HUVECs (5 × 10^5^ cells/well) were seeded in 6-well plates and left to adhere at 37 °C, 5% CO_2_, overnight, followed by a period of 24 h of starvation at serum-free medium. Cells were treated with 1 ml of DMEM supplemented with 10% FBS and either Dis*Ba*-01 (1000 nM), VEGF (10 ng/mL) or a co-treatment and incubated for 1 and 24 h at 37 °C, 5% CO_2_, followed by cell lysis. Twenty micrograms of protein from the cell lysate were separated on SDS-PAGE. Blots were probed with antibodies to **a** P-TY187 ERK1 + ERK2 and anti-ERK1 + ERK2; to **b** P-TY607 PI3K and anti-PI3K and GAPDH, this last used to normalize loading. Bands corresponding to all proteins were quantified by densitometry using the ImageJ FIJI program. Bar graph shows the mean ± SE of phosphorylated ERK1 + ERK2/ERK1 + ERK2/GAPDH and PI3K/PI3K/GAPDH expression from three independent experiments. Values of *p < 0.05 were significantly different when compared to untreated (a), Dis*Ba*-01 (b) and VEGF (c) groups

Activation of VEGF-A/VEGFR2 signalling cascade induces angiogenesis by promoting EC proliferation, survival, migration and morphogenesis. This occurs partially through the activation of the mitogen-activated protein kinase/extracellular-signal-regulated kinase-1/2 (ERK1/2) and partially through phosphatidylinositol-3-kinase (PI3K)/Akt signal transduction pathways [41]. We determined how Dis*Ba*-01 affects crucial VEGFR2 signalling pathways on VEGF-induced cells. VEGF increased ERK1/2 phosphorylation 1 h (33%) and 24 h (62%) after incubation compared with the unstimulated group (Fig. 5a). However, Dis*Ba*-01 decreased VEGF-induced ERK1/2 phosphorylation at 1 h (63.6%) and 24 h (87%). The phosphorylation status of PI3K was only altered by Dis*Ba*-01 on VEGF-induced cells after a 1-h treatment (36.6% of inhibition) (Fig. 5b). These results clearly illustrate that Dis*Ba*-01 sustained the angiogenesis inhibition at least by 24 h by blocking VEGFR2-mediated ERK1/2 signalling pathways.

### Dis*Ba*-01 changes F-actin organization in HUVECs

**Fig. 6:**
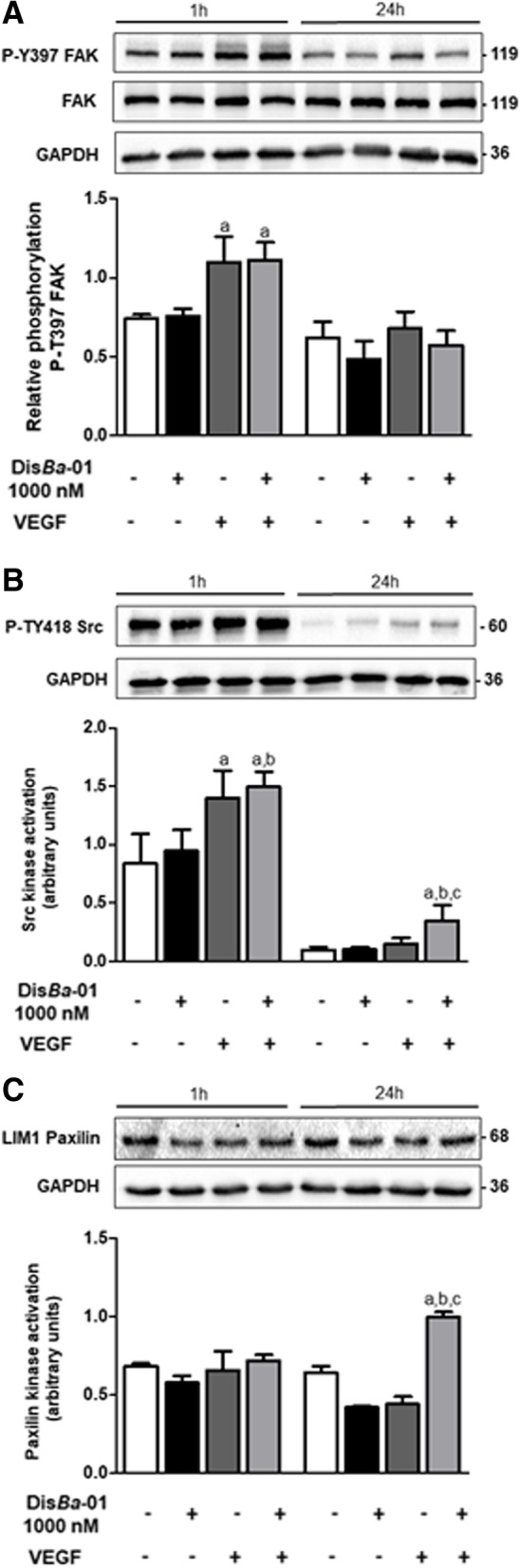
Dis*Ba*-01 promotes FAK, Src and paxillin phosphorylation. HUVECs (5 × 10^5^ cells/well) were seeded in 6-well plates and left to adhere at 37 °C, 5% CO_2_, overnight, followed by a period of 24 h of starvation at serum-free medium. Cells were treated with 1 ml of DMEM supplemented with 10% FBS and either Dis*Ba*-01 (1000 nM), VEGF (10 ng/mL) or a co-treatment and incubated for 1 and 24 h at 37 °C, 5% CO_2_, followed by cell lysis. Twenty micrograms of protein from the cell lysate were separated on SDS-PAGE. Blots were probed with antibodies to **a** P-Y397 FAK and anti-FAK; to **b** P-TY418 Src; to **c** phospho LIM1 Paxilin; and GAPDH, this last used to normalize loading. Bands corresponding to all proteins were quantified by densitometry using the ImageJ FIJI program. Bar graph shows the mean ± SE of phosphorylated pFAK/FAK/GAPDH, pSrc/GAPDH and pLIM1Paxilin/GAPDH expression from three independent experiments. Values of *p < 0.05 were significantly different when compared to untreated (a), Dis*Ba*-01 (b) and VEGF (c) groups

Endothelial cell adhesion to the ECM is mostly mediated by integrins, whose activation changes cytoskeleton proteins through the binding of signalling molecules such as FAK. FAK is a 125-kDa cytoplasmic tyrosine kinase protein found at adhesion sites responsible for activation of cell adhesion, motility and survival responses. Most importantly, FAK is the main transducer of the integrin-mediated signalling pathway required to stabilize the actin cytoskeleton, by creating a kinase complex with SrC which uses paxillin as a major substrate [42, 43]. Dis*Ba*-01 inhibited migration and changed the morphology of tubes, leading to the questioning of whether Dis*Ba*-01 could interfere with VEGF-mediated response in FAK/SrC/paxillin signalling. VEGF increased FAK and SrC phosphorylation after 1 h but Dis*Ba*-01 did not affect this response (Fig. 6a-b). Although SrC and paxillin phosphorylation remained unaffected by VEGF in 24 h, the phosphorylation of both proteins was increased by Dis*Ba*-01 in VEGF-induced cells at this time (Fig. 6b-c).

**Fig. 7:**
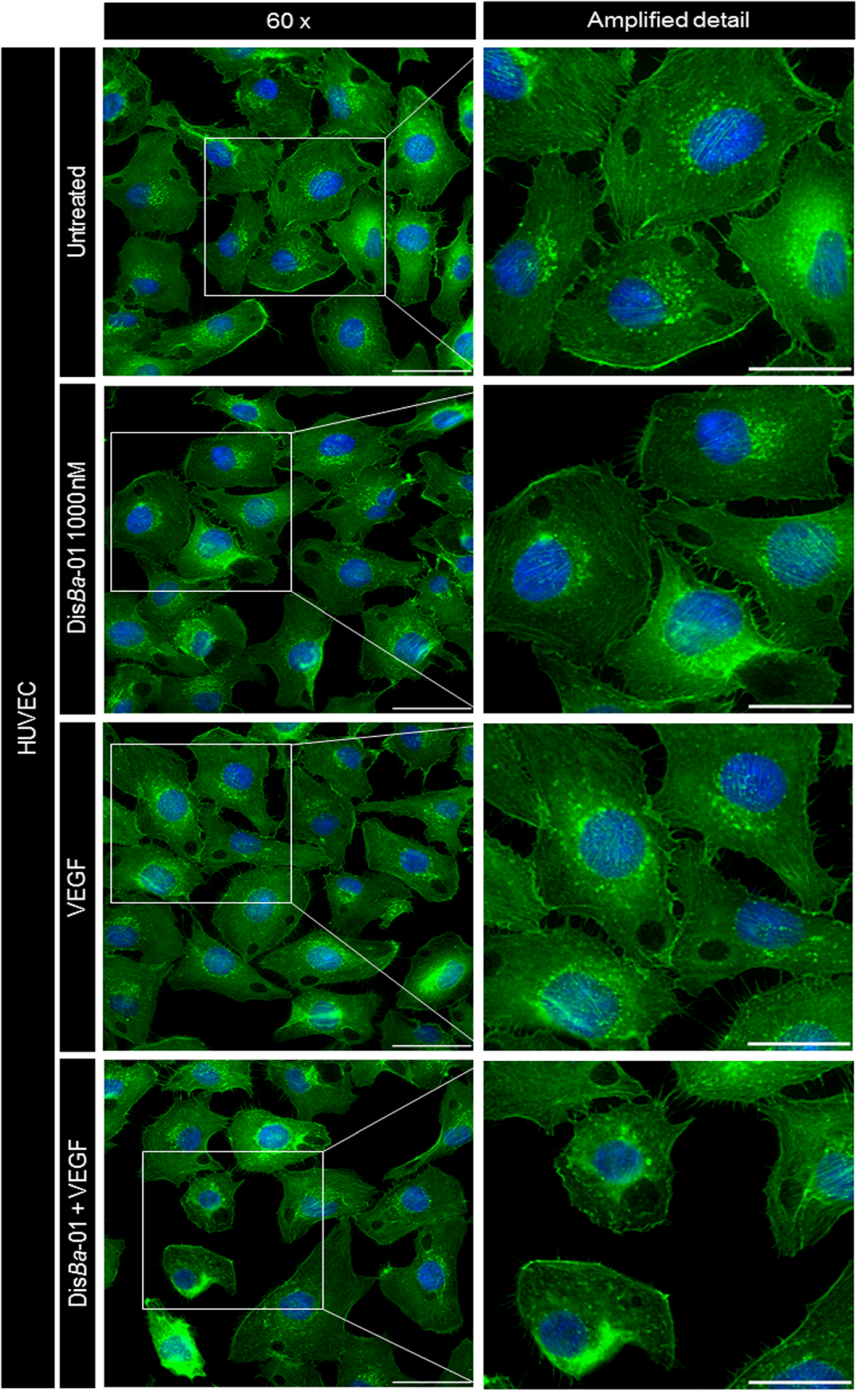
DisBa-01 induces morphological changes in endothelial cells. HUVECs (3 × 10^4^ cells/well) were plated in a 96-well microplate previously coated with FN (1 μg/cm^2^), in serum-free DMEM and incubated overnight at 37 °C, 5% CO_2_. Cells were exposed to VEGF (10 ng/mL), Dis*Ba*-01 (1000 nM) and VEGF plus Dis*Ba*-01 for 30 min in DMEM 10% FBS. Cell nuclei were stained with DAPI (0.7 ng/μl) and cytoplasm was stained with Alexa Fluor™ 488 phalloidin for 10 min. Images were observed with 60x magnification. Representative images were obtained from three independent experiments. Scale bar = 50 μm (left panel) and 20 μm (right panel)

In order to evaluate the morphological changes in HUVECs treated with Dis*Ba*-01 and/or VEGF, cells were stained using a fluorescent green probe to F-actin (phalloidin). VEGF treatment did not change cell morphology but Dis*Ba*-01 induced evident structural changes in the cell appearance (Fig. 7). Cells lose protrusions and acquire a circular format, as an indirect consequence of integrin blocking by DisBa-01, resulting in loose adhesions that in turn affect FAK/SrC/paxillin downstream signalling and actin re-organization. These events will contribute to impaired cell migration.

### Dis*Ba*-01 co-localizes with α_v_β_3_ and VEGFR2

**Fig. 8:**
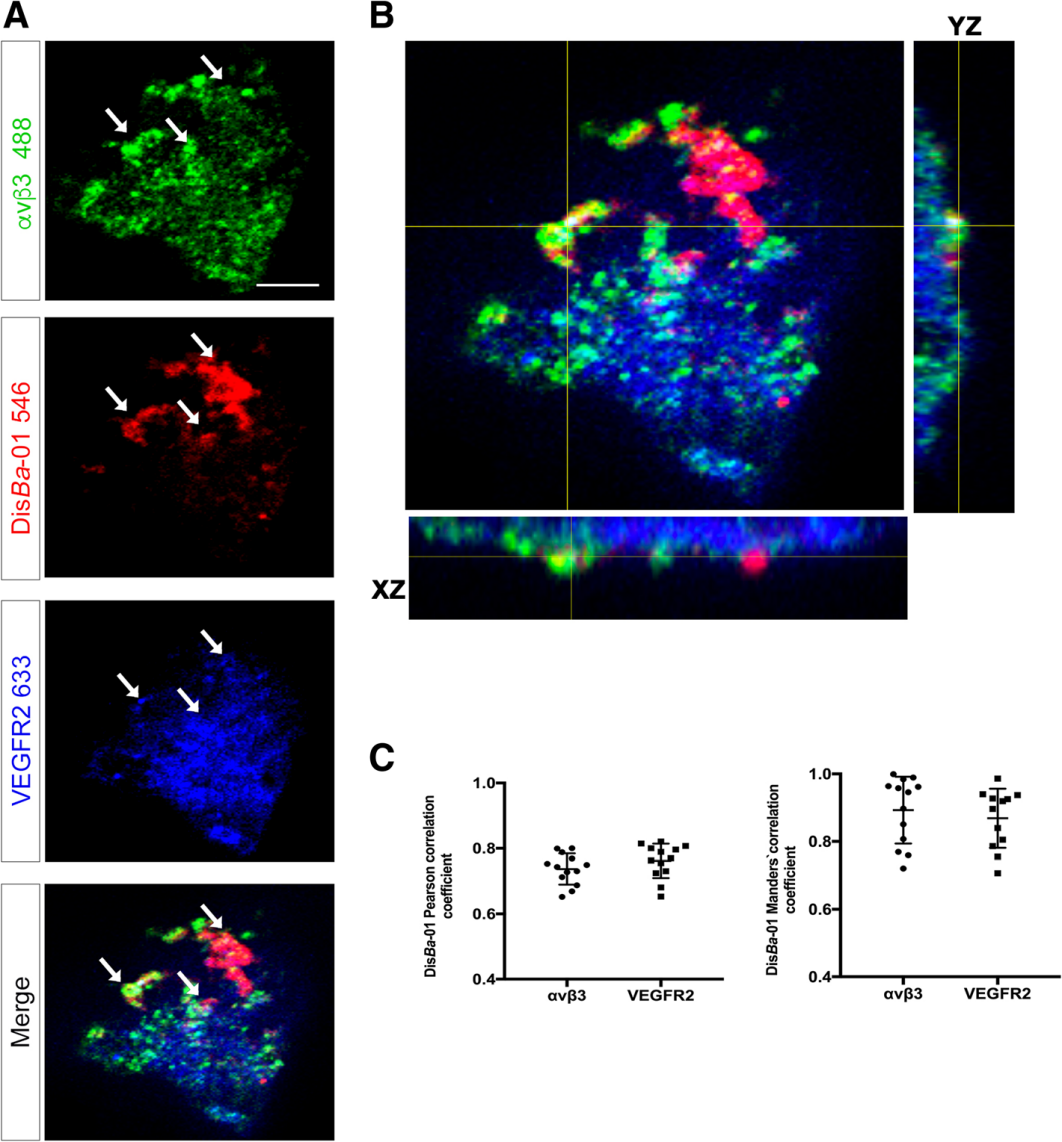
Dis*Ba*-01 colocalizes with VEGFR2 and α_v_β_3_. **a** Representative confocal images of triple stained HUVECs cells cultured in FN coated plates: αvβ3 (Green), Dis*Ba*-01 (Red), and VEGFR2 (Blue) in separated and merged channels. White = triple colocalization. **b** Orthogonal view of Z-stack projections showing the colocalization of αvβ3 integrin, Dis*Ba*-01 and VEGFR2 receptor. **c** Measurement of colocalization coefficients (tM1 and Pearson) of Dis*Ba*-01 with VEGFR2 and αvβ3. Results represent the average of *n* = 10 cells from three independent experiments. Slides were analyzed by confocal microscopy and pictures were taken using 63x magnification. Scale bar = 5 μm


We next tested if Dis*Ba*-01 co-localizes with α_v_β_3_ integrin and VEGFR2 during cell spreading in FN. The fluorescence signal for Alexa Fluor-546 labelled Dis*Ba*-01 (red color) was detectable on the cell surface 5 min after treatment (Fig. 8a-b and Additional file 2: Figure S2) and co-localizes with α_v_β_3_ (green color, Fig. 8a-b) and VEGFR2 (blue color, Fig. 8a-b and Additional file 3: Video S1). Mander’s and Pearson’s Colocalization Coefficients are represented in Fig. 8c.

**Additional file 3: Video S1** Three-dimension projection from confocal imaging of triple stained HUVECs cells cultured in FN coated slides, Related to Fig. 8. The movie was generated by Image J Fiji software from a Z-stack image (36 slices), and represents the whole cell and the superposition of αvβ3 (green), Dis*Ba*-01 (red), and VEGFR2 (blue) signals. (MP4 7372 kb)


## Discussion

In endothelial cells, the interaction between α_v_β_3_ integrin and VEGFR2 is of particular importance during vascularization. The cross-talk between these two receptors regulates several cellular activities involved in tumor angiogenesis, including maximal transduction of angiogenic growth factors, migration and survival of endothelial cells, and tube formation [13, 20, 44]. Disabling these interactions by compromising both components, α_v_β_3_ and VEGFR2, could improve the effectiveness of current anti-angiogenic strategies and potentially block one of the mechanisms that contributes to therapy resistance. Full understanding of the mechanism of action of integrin inhibitors may be helpful in the translation for clinical studies. Cilengitide, a RGD-cyclic peptide with nanomolar inhibitory activity to α_v_β_3_ and α_v_β_5_ integrins, has been tested in patients with various advanced solid tumors such as malignant gliomas, which are highly angiogenic [45, 46]. However, after 10 years of clinical trials with cilengitide, the results are still not favorable. The reasons for the lack of success may be related to the dose discrepancy, in which only high doses of this peptide have anti-angiogenic effects [47]. In addition, low concentrations (nanomolar scale) of cilengitide induce VEGF mediated angiogenesis by altering α_v_β_3_ integrin and VEGFR2 trafficking, thereby promoting endothelial cell migration and pro-angiogenic effects [48].

Here we present further evidence on the potent anti-angiogenic mechanism of Dis*Ba*-01, a RGD-disintegrin and α_v_β_3_ inhibitor, whose inhibitory effect on angiogenesis was previously described [35, 39], although its mechanism of action was not completely understood. Our data provide evidence for a key role of α_v_β_3_ integrin in controlling VEGF signaling by using a specific antagonist such as Dis*Ba*-01. This disintegrin inhibits VEGF-mediated angiogenesis by impairing α_v_β_3_/VEGFR2 cross talk. Dis*Ba*-01 inhibits several angiogenic cascade steps induced by VEGF, which includes proliferation, migration, invasion, adhesion and tube formation. As demonstrated by previous studies, RGD disintegrins, such as triflavin, accutin, salmosin, rhodostomin and contortrostatin, have similar anti-proliferative, anti-migratory, anti-adhesive and anti-angiogenic effects mediated or not by growth factors in endothelial cells [25, 49, 50]. However, none of these studies showed the occurrence of a synergistic inhibition between VEGFR2 and α_v_β_3_ integrin by RGD-disintegrins. Here we show that the presence of VEGF does not influence the inhibitory effect of Dis*Ba*-01 on HUVEC migration and invasion. Furthermore, DisBa-01 prevents the VEGF stimulatory effect on HUVEC proliferation. These results indicate that the integrin α_v_β_3_ dominates VEGF signaling. Similar findings were observed for plasminogen activator inhibitor-1 (PAI-1). This protein inhibits VEGF-induce VEGFR2 phosphorylation in HUVECs plated on vitronectin but not on fibronectin or collagen [51]. PAI-1 also inhibits the interaction between VEGFR2 and αvβ3 integrin as well the downstream signaling pathways after VEGF treatment. Interestingly, either DisBa-01 or VEGF increased cell adhesion to VN but not to FN. However, when the two proteins were associated, cell adhesion was decreased, although still higher than the control. The mechanism of this effect is not understood yet but it indicates an inhibitory cross-talk among VEGFR2 and αvβ3 integrin receptors.

A possible mechanism of action for the impairment of VEGFR2/α_v_β_3_ cross-talk by Dis*Ba*-01 is the modulation of expression and phosphorylation status of VEGFR2 and α_v_β_3_ integrin. VEGF activated endothelial cells by promoting VEGFR2 phosphorylation, and accumulation of internalized VEGFR2 in endosomes and lysosomes [52, 53]. Dis*Ba*-01 was able to attenuate VEGFR2 protein expression without affecting β_3_ integrin content. Cilengitide and S36578, another α_v_β_3_ antagonist, induced rapid recycling of internalized VEGFR2 and prevented VEGFR2 degradation, shuttling VEGFR2 back to the plasma membrane, thus amplifying the cellular response to VEGF [47]. These results are very distinct from the DisBa-01 effects; however, future assays must be done in order to understand what happens to VEGFR2 after disintegrin binding. In addition, we also show that Dis*Ba*-01 did not affect VEGFR2 and α_v_β_3_ mRNA levels, suggesting that its effects on VEGFR2 and α_v_β_3_ expression occur at a post-transcriptional level.

Tyrosine (Y^773^) phosphorylation of the β_3_ integrin subunit occurs in response to VEGF and it is essential for VEGFR2–β_3_ association, VEGFR2 activation and subsequent signaling. Thus, the cross-talk between the two receptors determines the cellular responses to VEGF, as well as the binding affinity of the integrin, which is regulated by tyrosine phosphorylation events [20, 54]. Antibody blockade to α_v_β_3_ integrin function inhibits VEGFR2 phosphorylation, indicating that this phosphorylation is α_v_β_3_ dependent. Similarly, VEGFR2 inhibitors impair the formation of complexes between VEGFR2 and β_3_ subunit [20, 54]. Here we show that Dis*Ba*-01 inhibits β_3_ phosphorylation induced by VEGF. One possible explanation for this effect is that disintegrin binding would induce integrin structural modifications that impair subsequent phosphorylation as previously suggested for RGD-cyclic peptides [47]. However, this hypothesis needs to be addressed in future studies.

Phosphorylation of β_3_ integrin subunit modulates several intracellular events, including VAV-1/Rho GTPase activation, actin cytoskeleton reorganization and regulation of the ERK1/2 and PI3K pathways, which are involved in the modulation of basic cellular functions such as cell spreading and survival [55, 56]. Additionally, a set of signaling kinases (ERK1/2 and PI3K) are also modulated via VEGFR2 activation by VEGF on HUVECs [9, 41, 57]. Our study demonstrated that Dis*Ba-*01 inhibits VEGF-dependent phosphorylation of ERK 1/2 and PI3K, suggesting that the inhibition of proliferation, migration and invasion might occur via EKR1/2 inhibition. Erk1 and Erk2 deletions in primary endothelial cells resulted in decreased cell proliferation and migration, impaired apoptosis and interestingly, inducing defects in the cytoskeleton organization, thus impairing cell motility [57, 58].

The importance of FAK as an antitumor endothelial target has been enhanced by the observation that its inhibition on endothelial cells prevented tumor metastasis, improving the function of the endothelial barrier [59]. Activated FAK exhibits phosphotyrosine anchor sites for several classes of signaling molecules, including those belonging to the PI3K/Akt signaling pathway [44] and Src [23, 24], which are involved in various cellular functions. Src is the major tyrosine kinase associated with β_3_ following stimulation of cells with growth factors and it is the possible kinase responsible for phosphorylation of β_3_ cytoplasmic tyrosines, a pathway that controls the functional association between α_v_β_3_ and VEGFR2, which, in turn, regulates activation of both receptors on ECs. This functional interplay is crucial for EC adhesion, migration and the start of the angiogenic programming in ECs [22, 60]. In the present study, we showed that Dis*Ba*-01 does not affect FAK or Src phosphorylation; however, FAK and Src activation by VEGF appears to be insufficient to fully activate downstream signaling pathways, such as ERK1/2 or PI3K. Another disintegrin, Kistrin, which selectively binds to α_v_β_3_ integrin, inhibits FAK/Src association and decreases cell response to VEGF [61].

Paxillin is a signal transduction adapter protein, associated with focal adhesions, and one of the main substrates of FAK. It has been reported that VEGF-A recruits FAK, which phosphorylates paxillin in ECs [62]. This phosphorylation induces the assembly of the paxillin-Crk-Dock180 molecular complex that regulates the activity of guanine-Rho triphosphatase and activates Rac in addition to extracellular signaling pathways regulated by kinase signals (ERK and Scr), leading to cell migration and adhesion [62]. Src phosphorylation induced by VEGF, in the presence of Dis*Ba*-01, was not enough to stimulate the ERK pathway and it did not result in paxillin activation, at least after 1 h treatment. Montenegro et al. [37], using oral squamous cancer cells (OSCC) treated with Dis*Ba*-01, showed an increase in paxillin immunostaining, justifying the presence of higher focal and maturity adhesions and a decrease in directionality and speed during cell migration. However, in this paper the authors incubated cells with DisBa-01 for longer periods (3 and 8 h), which may explain why we only see disintegrin effects after 24 h.

Previous studies demonstrated that phosphorylated VEGFR-2 co-immunoprecipitated with β3 integrin subunit, but not with β1 or β5, upon endothelial cell stimulation with VEGF-A [19]. More recently, total or phosphorylated VEGFR2 was demonstrated to co-immunoprecipitated with αxβ2 integrin [63] indicating that the two receptors interact physically. Here we demonstrated by confocal imaging that Disba-01 co-localizes with α_v_β_3_ integrin and with VEGFR2, although the characteristics of this interaction are not fully understood.

Finally, our data show different results from cilengitide, an integrin antagonist tested in clinical trials. Cilengitide (10 μM) increases α_v_β_3_ integrin affinity in endothelial cells leading to FAK activation, phosphorylation of Src and VE-cadherin [48]. DisBa-01 did not activate FAK or Src in our conditions; however, we used a much lower concentration, which makes any comparison difficult. In addition, the authors did not study the effect of cilengitide in the presence of VEGF nor VEGFR2-phosphorylation or its downstream ERK activation. Thus, our study brings new information on the mechanisms of integrin and growth factor receptors signaling.

## Conclusions

Even in the presence of exogenous VEGF, Dis*Ba*-01 impairs the α_v_β_3_ integrin/VEGFR2 intracellular signaling cross-talk in HUVECs, resulting in strong anti-angiogenic action and cellular morphological alterations. These results may be helpful to understand the effects of antiangiogenic drugs in clinical trials and may help develop new therapies against metastasis.

## Additional files

Additional file 1: **Figure S1.** Expression of β_3_ integrin under VEGF, DisBa-*01* or VEGF plus DisBa-*01* treatment. **(A)** Expression of β_3_ integrin subunit in HUVEC was analyzed by flow cytometry. The presence of αvβ3 integrin receptor on the cell surface was detected with FITC dye and specific antibodies (red curve) after 1 h treatment with Dis*Ba*-01 (1000 nM), VEGF (10 ng/mL) and co-treatment (Dis*Ba*-01 + VEGF). The black curve represents isotype control. **(B)** β_3_ mRNA (ITGB3) expression. HUVECs (5 × 10^5^/well) were plated in 6-well plates with DMEM and 10% FBS, followed by a 24-h starvation period on serum-free medium. Cells were then treated with Dis*Ba*-01 (1000 nM) and/or VEGF (10 ng/mL) for 24 h followed by lysis and RNA isolation. Quantitative RT-PCR was carried out using specific primers to human ITGB3 and GAPDH (housekeeping). Bar graph shows the mean ± SE of expression from three independent experiments. Values of *p < 0.05 were significantly different when compared to untreated (a), treated with Dis*Ba*-01 (b), and treated with VEGF (c). (TIF 1465 kb)

Additional file 2: **Figure S2.** Colocalization of αvβ3 with Dis*Ba*-01; VEGFR2 and Dis*Ba*-01 + VEGFR2. **(A)** Integrin α_v_β_3_ (green) and VEGFR2 (red) without Dis*Ba*-01 treatment. **(B)** Integrin α_v_β_3_ (green) and Dis*Ba*-01 (red). Yellow regions in merged image = double colocalization. **(C)** Integrin αvβ3 (green), Dis*Ba*-01 (red) and VEGFR2 (blue). Arrows indicate colocalization regions (yellow = double colocalization; white = triple colocalization. Scale bar = 5 μm. (JPG 1902 kb)

## Abbreviations

BCA: Bicinchoninic Acid Assay
CAFs: Cancer Associated Fibroblasts
DAPI: 4′,6-diamidino-2-phenylindole
Dis*Ba*-01: Disintegrin from *Bothrops alternatus*
Erk: Extracellular-signal-regulated kinase
FAK: Focal Adhesion Kinase
FITC: Fluorescein Isothiocyanate
HUVECs: Human Umbilical Vein Endothelial Cell
MMPs: Matrix Metalloproteinases
MTT: (3-(4,5-dimethylthiazol-2-yl)-2,5-diphenyltetrazolium bromide)
OSCC: Oral Squamous Cancer Cells
PAI-1: Plasminogen Activator Inhibitor-1
PI3K: Phosphatidylinositol 3-Kinase
Rac: Ras-related C3 botulinum toxin substrate
Rho: Ras homologous
Sdc-1: Syndecan-1
Src: non-receptor protein tyrosine kinase
TAMs: Tumor Associated Macrophages
VAV-1: Vav guanine nucleotide exchange factor 1
VEGF: Vascular Endothelial Growth Factor
VEGFR2: VEGF type 2 receptor
