# Supplementary figures and images for: Recombinant RGD-disintegrin DisBa-01 blocks integrin αvβ3 and impairs VEGF signaling in endothelial cells
Source: Cell Commun Signal. 2019 Mar 20;17:27. doi: 10.1186/s12964-019-0339-1 (PMC6425665; doi:10.1186/s12964-019-0339-1)

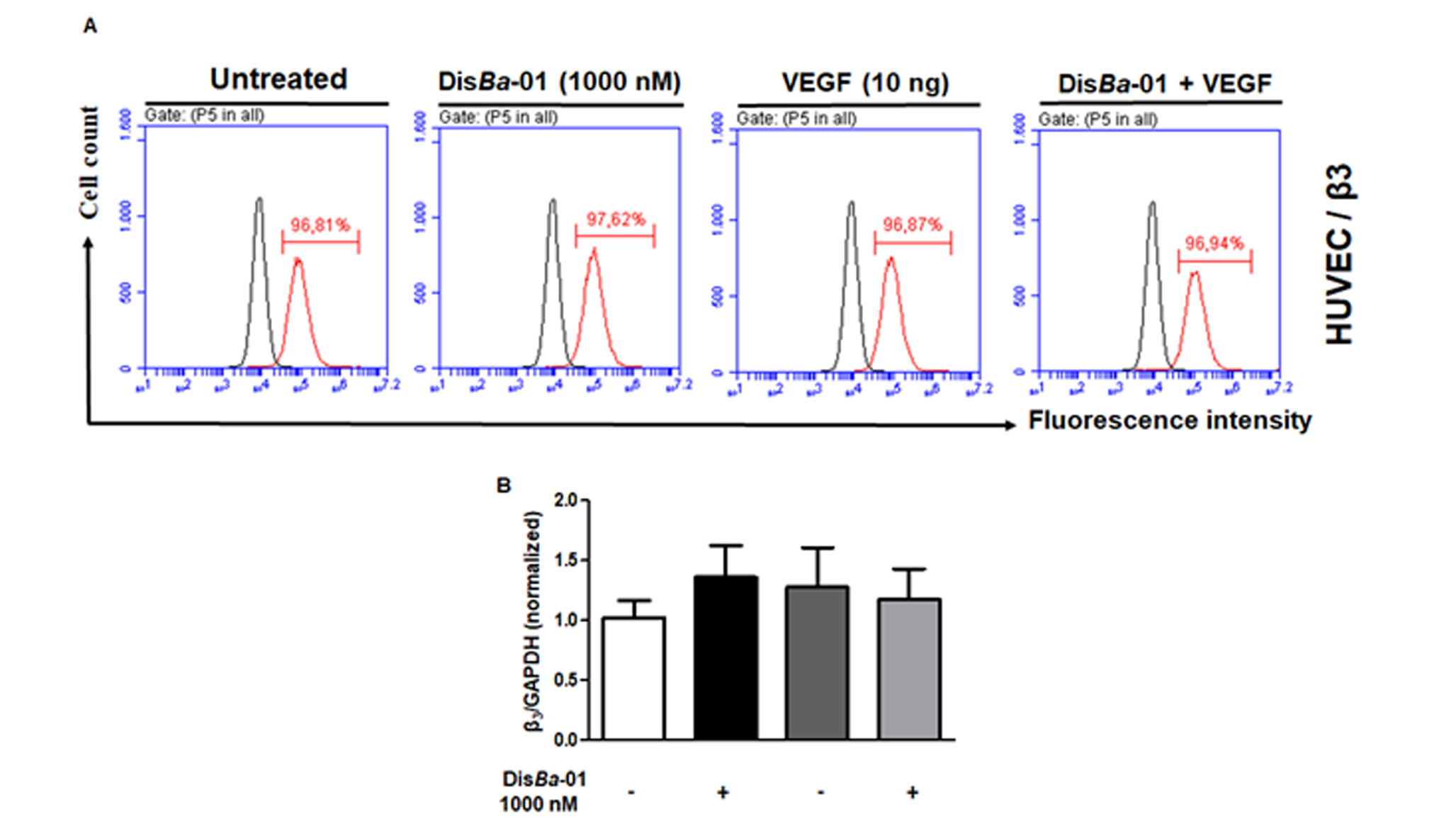

Supplement: Supplementary file 2 — Figure S1. Expression of β3 integrin under VEGF, DisBa-01 or VEGF plus DisBa-01 treatment. (A) Expression of β3 integrin subunit in HUVEC was analyzed by flow cytometry. The presence of αvβ3 integrin receptor on the cell surface was detected with FITC dye and specific antibodies (red curve) after 1 h treatment with DisBa-01 (1000 nM), VEGF (10 ng/mL) and co-treatment (DisBa-01 + VEGF). The black curve represents isotype control. (B) β3 mRNA (ITGB3) expression. HUVECs (5 × 10^5^/well) were plated in 6-well plates with DMEM and 10% FBS, followed by a 24-h starvation period on serum-free medium. Cells were then treated with DisBa-01 (1000 nM) and/or VEGF (10 ng/mL) for 24 h followed by lysis and RNA isolation. Quantitative RT-PCR was carried out using specific primers to human ITGB3 and GAPDH (housekeeping). Bar graph shows the mean ± SE of expression from three independent experiments. Values of *p < 0.05 were significantly different when compared to untreated (a), treated with DisBa-01 (b), and treated with VEGF (c). (TIF 1465 kb) [file 12964_2019_339_MOESM1_ESM.tif]

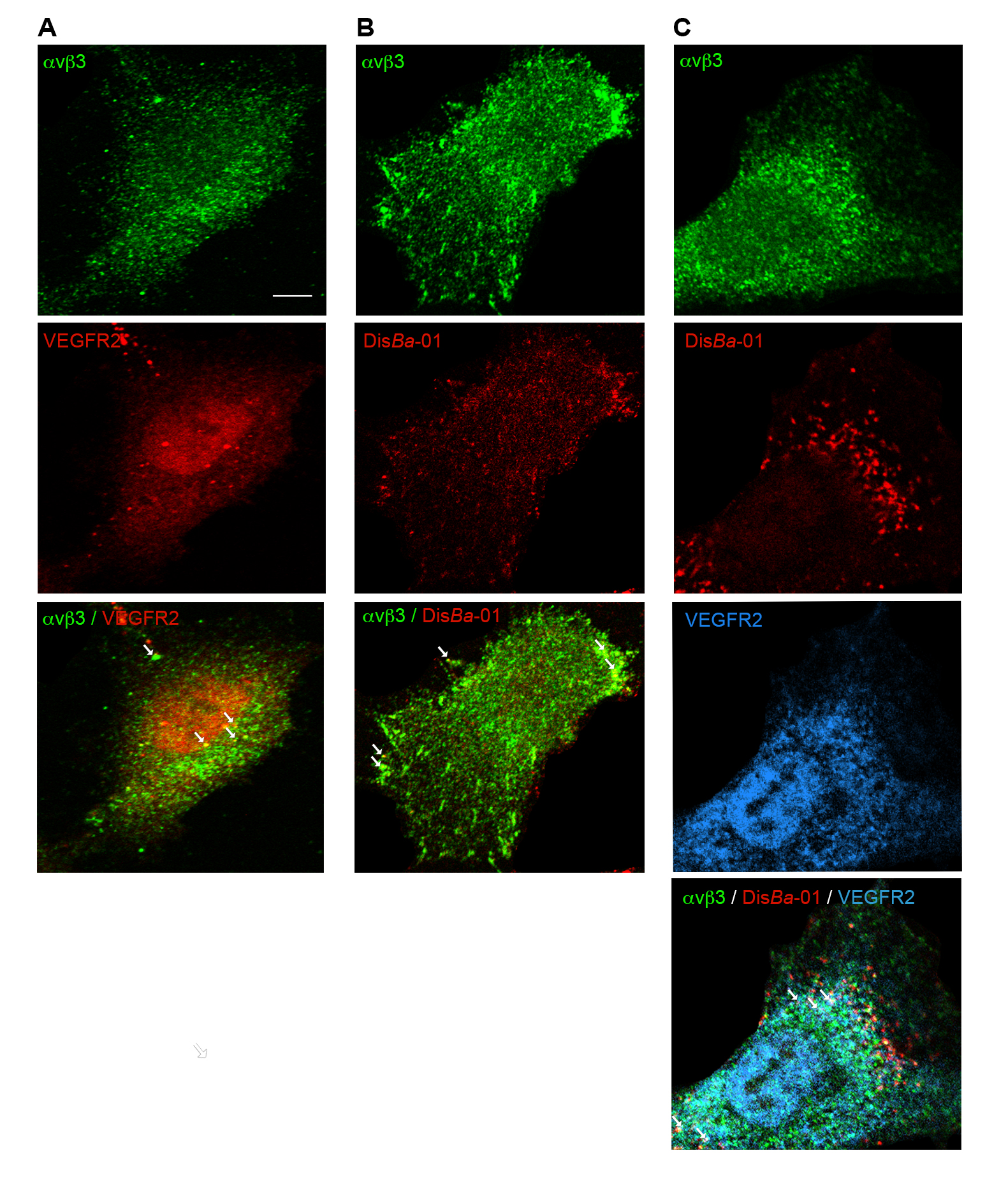

Supplement: Supplementary file 3 — Figure S2. Colocalization of αvβ3 with DisBa-01; VEGFR2 and DisBa-01 + VEGFR2. (A) Integrin αvβ3 (green) and VEGFR2 (red) without DisBa-01 treatment. (B) Integrin αvβ3 (green) and DisBa-01 (red). Yellow regions in merged image = double colocalization. (C) Integrin αvβ3 (green), DisBa-01 (red) and VEGFR2 (blue). Arrows indicate colocalization regions (yellow = double colocalization; white = triple colocalization. Scale bar = 5 μm. (JPG 1902 kb) [file 12964_2019_339_MOESM2_ESM.jpg]
